# Development and Characterization of High Performance Shape Memory Alloy Coatings for Structural Aerospace Applications

**DOI:** 10.3390/ma11050832

**Published:** 2018-05-18

**Authors:** Dimitrios A. Exarchos, Panagiota T. Dalla, Ilias K. Tragazikis, Konstantinos G. Dassios, Nikolaos E. Zafeiropoulos, Maria M. Karabela, Carmen De Crescenzo, Despina Karatza, Dino Musmarra, Simeone Chianese, Theodore E. Matikas

**Affiliations:** 1 Department of Materials Science and Engineering, University of Ioannina, 45110 Ioannina, Greece; dexarch@cc.uoi.gr (D.A.E.); pan.dalla@yahoo.gr (P.T.D.); itragazik@cc.uoi.gr (I.K.T.); nzafirop@cc.uoi.gr (N.E.Z.); mariakarab@gmail.com (M.M.K.); matikas@cc.uoi.gr (T.E.M.); 2 Department of Civil and Building Engineering, Design and Environment, University of Campania “L. Vanvitelli”, 81031 Aversa (CE), Italy; carmendecrescenzo@virgilio.it (C.D.C.); karatza@irc.cnr.it (D.K.); Dino.MUSMARRA@unicampania.it (D.M.); simeone.chianese@unicampania.it (S.C.)

**Keywords:** shape memory alloys (SMAs), coating, Ni–Ti, dfferential scanning calorimetry (DSC), shape memory effect (SME)

## Abstract

This paper presents an innovative approach, which enables control of the mechanical properties of metallic components by external stimuli to improve the mechanical behavior of aluminum structures in aeronautical applications. The approach is based on the exploitation of the shape memory effect of novel Shape Memory Alloy (SMA) coatings deposited on metallic structural components, for the purpose of relaxing the stress of underlying structures by simple heating at field-feasible temperatures, therefore enhancing their structural integrity and increasing their stiffness and rigidity while allowing them to withstand expected loading conditions safely. Numerical analysis provided an insight in the expected response of the SMA coating and of the SMA-coated element, while the dependence of alloy composition and heat treatment on the experienced shape memory effect were investigated experimentally. A two-phase process is proposed for deposition of the SMA coating in an order that induces beneficial stress relaxation to the underlying structure through the shape memory effect.

## 1. Introduction

The closely related properties of shape memory and pseudoelasticity in SMAs result from a reversible transformation from austenite to martensite and vice versa under applied mechanical load and/or temperature variations [1,2,3,4]. The parent austenite phase transforms to martensite upon cooling, which is dependent on alloy composition, material processing, and stress levels [5]. In the absence of mechanical load, the variants of the low-symmetry martensite phase are usually arranged via twinning in self-accommodation, leading to no obvious macroscopic change in shape. However, under sufficient mechanical loading only favorably oriented martensite variants form, leading to large macroscopic inelastic strains [6]. During heating, martensitic phase reverse transforms back to austenitic Ni-Ti phase, and the inelastic strains are recovered, a phenomenon evidenced as shape memory effect [6]. If a mechanical load is applied to the material in the twinned martensitic phase, detwinning is possible by reorientation of a certain number of variants [6]. The high inelastic strains associated with detwinning are not recovered upon removal of the load, while plastic deformation can take place upon continued loading [6].

Although a plethora of SMA-related work exists in the literature, never has the shape recovery characteristic of such alloys been utilized in coating applications with the purpose of relaxing the deformations experienced by underlying metallic structures, hence improving their structural integrity. This is the objective of the present work, wherein a new technology is presented for increasing the stiffness and rigidity of such structures while allowing them to withstand expected loading conditions safely to enhance the integrity of damaged structures and protect them from corrosion.

The SMA material of choice is binary Ni-Ti, since the specific material system exhibits strong shape memory effect and pseudoelastic behavior combined with excellent deformation behavior, high fatigue resistance, and resistance to corrosion. Moreover, the crystallography and thermomechanical response of Ni-Ti are well understood, as are the effects of heat treatment and the variation of transformation temperatures with changes in composition. In addition, Ni-Ti alloys are advantageous over conventional alloys with respect to corrosion resistance, since they do not require mixing with other compounds, such as in the case of corrosion-prone alloys (e.g., ferrous alloys), in which mixing with copper would offer the desirable anti-corrosive characteristic. Additionally, Ni-Ti SMAs allow tailoring of their phase transformation temperatures by appropriate heat treatment, which induces the formation of precipitates in their microstructure. Precipitates are non-transforming compounds, which shift the phase transformation temperatures, decrease the phase transformation strain, and suppress the Transformation Induced Plasticity (TRIP) [1,3,7,8,9,10,11,12,13].

SMAs with two different Ni-Ti compositions are considered, namely, equiatomic and Ni_50.8_Ti_49.2_ (at %) alloys. While Ni-Ti in equiatomic composition does not form precipitates when heat treated, Ni-rich alloys such as Ni_50.8_Ti_49.2_ (at. %) SMA form Ni_4_Ti_3_ precipitates when aged [14]; the precipitates grow in size with raising temperature and heating duration. Ni_4_Ti_3_ nano-precipitates start to form alongside grain boundaries at smaller aging heat treatment temperatures and durations. Higher aging temperatures or durations lead to the growth of the size of precipitates, which start to spread homogeneously across the matrix. With further aging, in terms of either temperature or time, the precipitates continue to grow in size. Therefore, raising aging temperature and duration causes evenly distributed, fine, coherent-with-the-matrix precipitates to form large incoherent precipitates. Because Ni_4_Ti_3_ particulates are rich in nickel, their precipitation reduces the nickel content in the matrix, therefore making it richer in titanium in comparison to the original (without precipitates) material. This strengthens the SMA and increases the transformation temperatures, because the nickel content in the matrix decreases [15]. In addition, Ni_4_Ti_3_ precipitates play an important role in the two-way shape memory effect (SME) [16]. 

In this work, the expected thermomechanical responses of the SMA coating and of the SMA-coated metallic element as a whole system were analyzed numerically by establishment and implementation of a thermomechanical SMA model, presented in Section 2. Ni-Ti alloy materials with different compositions have been considered in this study. These materials were subjected to various aging heat treatments to alter their microstructure by introducing precipitates of different sizes. Section 3 discusses the Ni-Ti material processing and coating deposition process in order to obtain a shape memory effect. The influence of the Ni-Ti composition and precipitate formation on the thermomechanical properties of the material is presented in Section 4. Based on the investigation of the effect of different heat treatments on the shape memory effect of the material, a two-phase coating deposition process is proposed for the SMA coating to exhibit shape memory effect, which will be beneficial to the partial relaxation of stress in the underlying structure. The SMA coatings developed herein on aluminum structural elements are targeted for aeronautical engineering applications, as, for example, in the aircraft wing box structure reinforcement and the fuselage structure to enhance their structural integrity.

## 2. Numerical Analysis

Numerical analysis of the SMA coating and of the SMA-coated structure is performed to gain insight into its expected thermomechanical response. For this purpose, the unified constitutive model for polycrystalline SMAs proposed by Boyd and Lagoudas [3] is implemented into ABAQUS software suite (6.12, Dassault Systèmes Simulia Corp., Providence, RI, USA) via a user material subroutine. The model is established based on continuum thermodynamics adopting the classical small-strain rate-independent flow theory for equations of evolution of transformation strain [1]. 

In the context of elastic isotropic response, increments of the components of strain tensor, *dε_ij_*, are given as

(1) $$d\varepsilon_{ij}^{t}=\Lambda_{ij}d\xi ,\;\Lambda_{ij}=\{\begin{array}{l} \Lambda_{ij}^{f},\;d\xi >0\\ \Lambda_{ij}^{r},\;d\xi <0 \end{array}$$

in which *ξ* is the martensitic volume fraction evolution and Λ_*ij*_ are the components of the direction tensor, defined as

(2) $$\Lambda_{ij}^{f}=\frac{3}{2}\frac{H^{cur}}{\bar{\sigma }}s_{ij},\;\Lambda_{ij}^{r}=\frac{\varepsilon_{ij}^{t}}{\xi }.$$

in which $H^{cur}$ is the uniaxial transformation strain magnitude for the complete transformation, $\bar{\sigma }=\sqrt{\frac{3}{2}s_{ij}s_{ij}}$ is the Mises equivalent stress, and $s_{ij}=\sigma_{ij}-\sigma_{kk}\delta_{ij}/3$ are the stress deviator constituents.

The developed constitutive model was employed in ABAQUS (6.12, Dassault Systèmes Simulia Corp., Providence, RI, USA). The highly non-linear response of SMAs requires the utilization of a non-linear FE analysis procedure. The FE analysis procedure is discretized in a finite number of time steps in order to precisely capture the material response. Additionally, because of the appearance of the time derivatives of the external material state variable σ and internal material state variable ξ, the analysis is not only non-linear but also transient. Hence, each analysis step corresponds to a physical time step with resolution Δt, with each beginning at time t and ending at time t+Δt. The Newton-Rapson (N-R) iterative-incremental procedure is utilized by the ABAQUS global solver at every time step of the analysis for the solution of the FE problem and an implicit time integration technique.

Provided that the analysis procedure is partitioned into time steps, throughout the rest of this discussion the values of the terms referred to the beginning of each time step will be denoted with the superscript t at the end of the time increment with the superscript t+Δt. At the beginning of each time step, the value of $\varepsilon_{t+\Delta t}$ is known based on the guesses of the N-R process regarding the solution variables and is input to UMAT subroutine as follows

(3) $$\varepsilon_{t+\Delta t}=\varepsilon_{t}+\Delta \varepsilon ,$$

in which Δε is the increment of the respective variable in the current time step.

### 2.1. Modeling the Structure: Compressive Stresses Induced in the Elastic Structure from the SMA Coating

The effectiveness of the implemented constitutive law in the combined SMA coating and underlying structure performance is assessed in this section. Since aircraft structures such as the aircraft wing box structure reinforcement and the fuselage are modular structures constructed of beams, the study considers a single beam structure. Such SMA coating applications entail, therefore, a long prism of aluminum with their lateral surfaces encased in SMA coating. 

A long rectangular aluminum beam with its lateral surfaces encased in SMA coating is considered. The dimensions are defined as follows: $l_{x}$, $l_{y}$, and $l_{z}$ represent the dimensions of the elastic structure, as they correspond to the respective coordinate axes, and $t_{SMA}$ represents the thickness of the SMA coating. Note that the SMA coating is encased only on the free surfaces of the beam perpendicular to the y-direction. $L_{x}$ and $L_{z}$ denote $l_{x}+t_{SMA}$ and $l_{z}+t_{SMA}$, respectively. For the adopted geometry $l_{x}=4$ mm, $l_{z}=4$ mm, $l_{y}=40$ mm, and $t_{SMA}=0.01$ mm, which yields an SMA coating ratio of 1:400 (the ratio of the thickness of the SMA coating to the smallest dimension of the matrix in the x−z plane). Two more geometries are considered with SMA coating ratios of 1:50 and 1:10. For these geometries, either (i) the longitudinal length and the SMA-coating thickness remain unchanged while the thickness of the elastic structure changes or (ii) the longitudinal length and the elastic structure thickness remain unchanged while the SMA-coating thickness changes. 

Two different loading paths are considered in the analysis, namely:

1. The SMA coating is assumed initially in the austenitic phase, and a uniaxial tensile load is applied in the longitudinal direction (y-direction) sufficient for phase transformation from austenite to martensite, followed by a subsequent heating sufficient to induce reverse transformation back to austenite;
2. The SMA coating is oriented martensite in the longitudinal direction, i.e., there is a macroscopic strain that can be recovered upon heating that will induce phase transformation from martensite to austenite.

If the dimensions of the beam are orders of magnitudes greater than the thickness of the SMA coating, which is the case for the geometry at hand—and should be the case in most structural applications involving coatings—then proper discretization using 3-D elements may lead to millions of finite elements, which renders a numerical solution intractable. One way out is the use of shell elements for simulating the SMA coating. Here, a different approach is adopted. Actually, both approaches were tested and found to yield results that differ by less than 2%. Periodic boundary conditions are assumed on a thin (in the y-direction) slice of the structure. To further simplify the calculations, only one quarter of the geometry is considered, and symmetry conditions are applied. This geometry and boundary conditions approximate the mechanical fields of the original problem away from the boundaries in y-direction (y=0, and $y=l_{y}$) as long as the longitudinal length $l_{y}$ is a lot bigger than the other dimensions of the structure. The simplicity of the geometry allows imposition of periodic boundary conditions by fixing of the bottom faces of both the aluminum beam and the SMA in the y-direction and by constraining the top faces of both materials to remain planar and parallel to the bottom faces. Rigid body motion is suppressed by pinning the node on the bottom surface of the elastic structure at the center of the full geometry (at the corner of the quarter geometry that is farthest away from the SMA coating), thus constraining the displacement of the node in all three coordinate directions. To apply symmetric boundary conditions, the negative *x*-faces and negative *z*-faces of both the matrix and the SMA are constrained with *x*-symmetry and *z*-symmetry constraints, respectively, thus fixing their displacement along their normal axis and their rotation along their two planar axes. 

The properties of the chosen SMA [1] for the numerical simulations presented here are shown in Table 1, and those of the aluminum beam are shown in Table 2.

**Figure 1:**
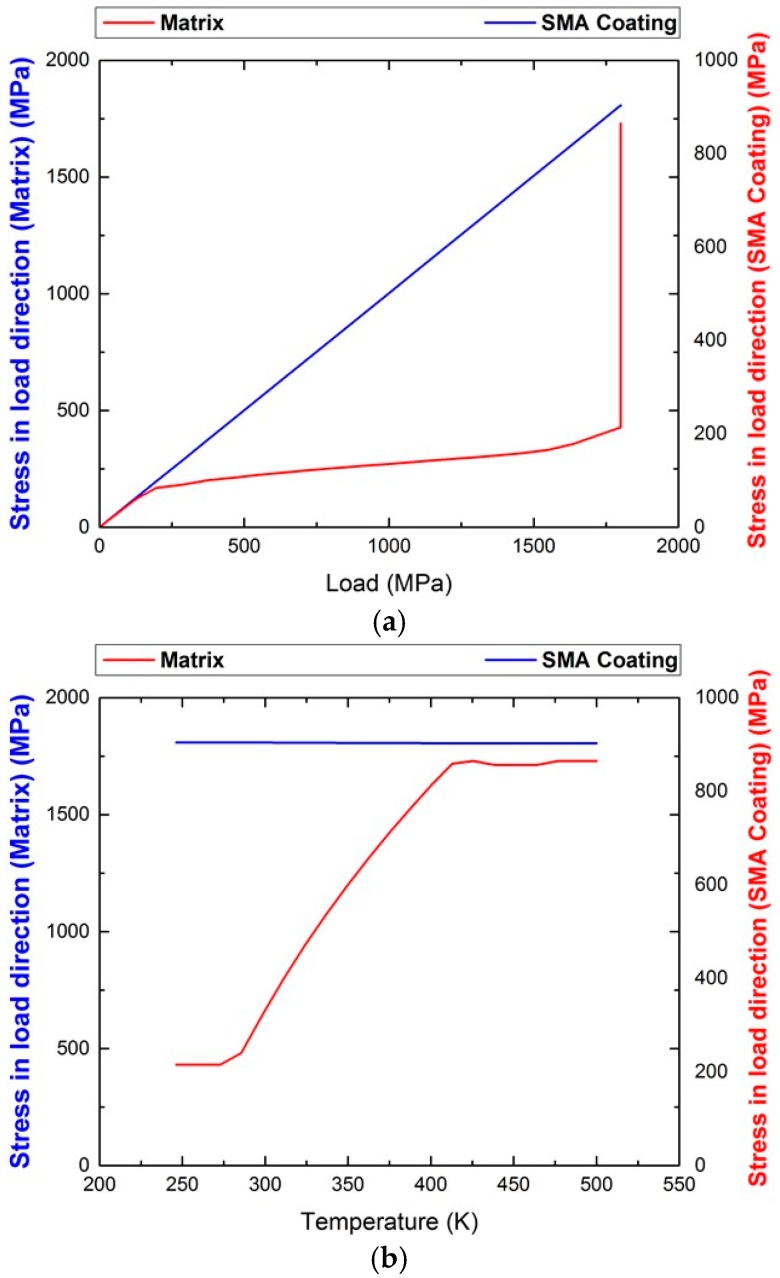
Normal stress in the longitudinal direction σ_yy_ (**a**) as a function of applied load and (**b**) as a function of temperature (K).

For the first loading path, wherein the SMA-coating deforms together with the elastic structure under an applied uniaxial load in the y-direction, the volume ratio between the SMA-coating and the aluminum beam is such that although the temperature at the application of the load is just 3 degrees above the martensitic start temperature $M_{s}$, the stiffness of the elastic structure requires a stress level of almost 1.8 GPa for the phase transformation in the coating to finish (Figure 1a). The reason being that the transformation strains of the coating should be matched by the elastic deformation of the beam. Subsequent heating of the whole structure reduces the levels of stress in the elastic structure negligibly (Figure 1b). These results are of course dependent on the elastic properties of the two phases; however, in any case, the required load levels for the transformation strain to take values above 0.01 in the SMA coating for this geometry are extremely high for practical applications. Thus, it seems that this loading path cannot be beneficial for the aluminum beam.

**Figure 2:**
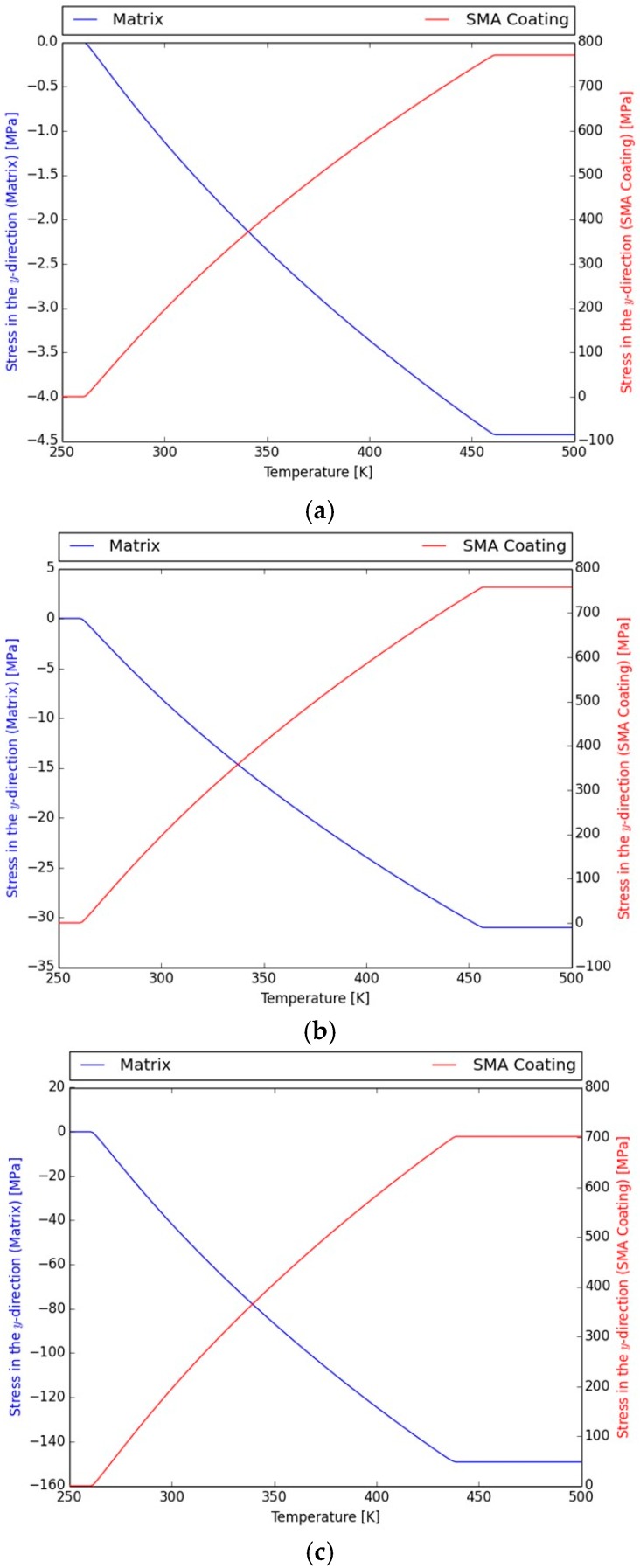
Normal stress in the longitudinal direction σ_yy_ as a function of temperature (K) for both the matrix and the SMA coating (**a**) 1:400 model, (**b**) 1:50 model, and (**c**) 1:10 model.

An alternative loading path is considered in which the SMA-coating is assumed to be in an oriented (in the y-direction) martensitic state at zero load and the whole structure is heated for contraction in the coating to take place by reverse phase transformation and compressive stresses to develop in the elastic structure. In Figure 2a, the evolution of the normal component of the stress tensor in the y-direction, i.e., $\sigma_{yy}$ during heating, is presented in both the SMA coating and the elastic structure. Here, stresses are observed to be almost uniform, except for a very small region at the interface between the two phases. The initial strain in the SMA before heating, i.e., in the martensite state, is $\varepsilon_{yy}=0.025$, and the stress is null both in the SMA and the aluminum beam. During heating, and as the SMA starts transforming into austenite recovering its initial deformation, the stress level in the SMA increases (tensile stresses develop), resulting in a compressive stress state in the elastic structure. The increase of stress in the SMA coating shifts the required temperature for further reverse transformation from martensite to austenite. The ratio of the thickness of the coating to the smallest dimension of the aluminum beam is such that the experienced tensile stress, once the coating is fully transformed into austenite, reaches as high as 780 MPa. Such stress levels require heating of around 200 K for the initiation to the end of phase transformation. At the end of phase transformation, the compressive stress value in the beam is a low as 4 MPa. These results are expected from a rule of mixtures perspective, since the load carrying capacity of the SMA coating in the modeled structure of a ratio 1:400 is quite limited.

Next, the geometry is altered, namely, the ratio of the SMA thickness to smallest dimension of the aluminum beam is changed to 1:50, and the levels of compressive stresses that can be developed in the elastic structure are evaluated (Figure 2b). For this geometry, the compressive stresses developed in the beam once the coating is fully transformed to austenite reach approximately 30 MPa, while the tensile stress level in the coating is around 750 MPa. As in the previous case, a temperature raise of approximately 200 K is required for full phase transformation. Lastly, for a ratio of 1:10, the compressive stresses developed in the beam once the coating is fully transformed to austenite is approximately 100 MPa, while the tensile stress level in the coating is around 700 MPa (Figure 2c). The temperature raise needed for full transformation is around 180 K.

### 2.2. Modelling the System SMA-coated Structure

Since SMA coatings on aluminum structural elements are being developed for modular structures made of beams; this section considers the benefits that a SMA coating can induce to the bending response of an aluminum beam. For the analysis, a straight beam of length l=40 mm and circular cross section is considered. The beam consists of an aluminum core of radius *r* and an SMA coating of thickness $t_{SMA}=0.01$ mm. Initially, the SMA coating is at an axially pre-strained martensitic phase, while the entire structure is under a uniform temperature of 250 K. To investigate the effect of the SMA coating on the response of the structure, three different geometries with ratios $r/t_{SMA}$: (i) 10, (ii) 40, and (iii) 400 are considered. 

**Figure 3:**
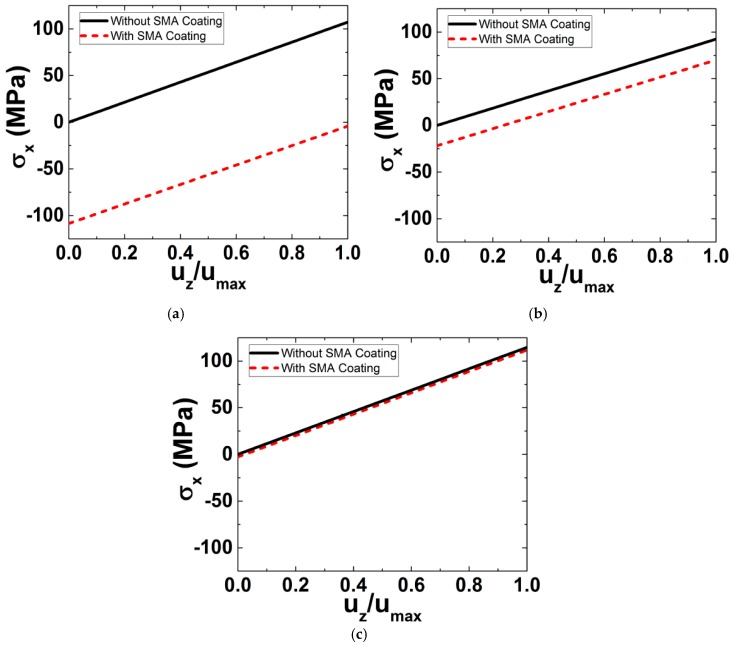
Maximum tensile stress on the aluminum surface *vs* normalized deflection for geometries with different $r/t_{SMA}$ ratios: (**a**) $r/t_{SMA}$ = 10; (**b**) $r/t_{SMA}$ = 50; (**c**) $r/t_{SMA}$ = 400.

The maximum stress values on the upper surface of the aluminum structure, which is under tension upon the application of the moment M_Y_, are shown in Figure 3 as a function of normalized deflection for each of the considered ratios $r/t_{SMA}$. Results for structures with and without SMA coatings are included in order to facilitate conception of the benefits of the SMA coating. The outcome of the simulations is that the tensile stresses induced in the presence of the SMA coating are reduced by an amount that is approximately equal to the initial compressive stresses throughout the bending loading. These compressive stresses thus contribute to the rigidity and stiffness of the aluminum beam to endure the applied loading conditions in a safe manner and are further expected to enhance the beam’s fatigue properties.

In Figure 3, the black line (same in the three plots) represents the reference (i.e., the behavior of the uncoated aluminum plate). Let us now observe the red dashed line. In Figure 3a, in which the coating thickness is too small compared to the substrate thickness (substrate/coating thickness ratio = 400), the behavior of the coating/aluminum system is similar to the uncoated aluminum (i.e., the coating has no effect). In Figure 3b, in which the coating becomes thicker (substrate/coating thickness ratio = 50), the coating starts to have an effect on the entire system. However, in Figure 3c, in which the coating thickness is only ten times less than the aluminum beam thickness, the effect of coating is very significant. From the analytical results presented in Figure 3, it can be concluded that the substrate/coating thickness ratio should be at least 50 for the SMA coating to have an effect on the entire structure.

## 3. Materials Processing and Characterization

In order to enable the Ni-Ti material to be beneficially used as SMA coating, Ni-Ti alloys of different compositions were considered, as well as the effect of different microstructures, producing precipitates of different sizes. The influence of the composition and precipitates on the thermomechanical properties was also examined. Two different Ni-Ti compositions were considered:

(a) Equiatomic Ni-Ti composition, which has fixed transformation metrics and high transformation strain and is prone to large amounts of TRIP, since there are no precipitates to stabilize the response [1,17];
(b) Ni-rich Ni_50.8_Ti (at %) composition, in which precipitates can be formed by appropriate heat treatment, thereby stabilizing the response of the material against TRIP. This composition offers tunable transformation metrics and adequate resistance to TRIP but lower transformation strains [1,17].

The following rationalization facilitates conception of the effect of precipitates on TRIP. During phase transformation, there is deformation misfit between the martensitic and austenitic phases present in the material. At that instance, the stress can locally exceed the material’s yield strength, leading to local plasticity. By inducing obstacles such as precipitates to the movement of dislocations, one can obtain less TRIP. Therefore, precipitates, as non-transforming particles, cause transformation strains to decrease (by amounts as per the rule of mixtures) [18]. In the case of Ni-Ti SMA with equiatomic composition, in absence of precipitates, there is more plasticity in the material, but also transformation strains are higher. In the Ni-rich material, the presence of precipitates causes suppression of plastic deformation [6]. Hence, if an application requires using an SMA material in a large number of cycles, as for example in an actuating device, the Ni-Ti composition of choice must be Ni-rich. On the other hand, applications requiring the use of the SMA material for a few cycles, as for example the SMA coating structural application of the present study, both equiatomic, as well as Ni-rich, SMAs can be equally used.

**Figure 4:**
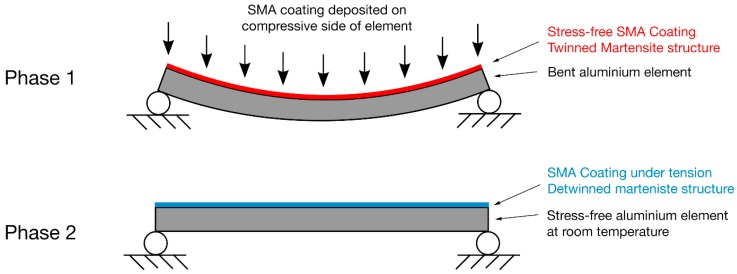
SMA coating deposition process on an aluminum substrate in order to for the coating to obtain the SME.

In this study, Ni-rich Ni_50.8_Ti was selected as the material of choice for the target coating application, i.e., aeronautical structural components anticipated to undergo a number of fatigue cycles. In addition to Ni-Ti composition, the geometry of the aluminum substrate necessary for deposition of the coating plays an important role in an effort that the SMA coating obtains a shape memory effect, which is beneficial towards the partial mitigation of stress on the underlying structure (Figure 4). To this regard, a two-phase coating deposition process is being proposed. During Phase 1, the SMA coating is deposited on the compressive side of a bent aluminum substrate. After deposition of the SMA coating, the coating/substrate structure will be left to reach ambient temperature, while the structure remains bent, so that the Ni-Ti coating reaches the martensitic state. Since, at this stage, no stress is applied on the coating, the SMA coating material is self-accommodated matrensite—twin structure. This completes Phase 1. During Phase 2, the SMA coating/substrate structure will be unloaded, regaining its original shape. The SMA coating then will be under tension in a detwinned state. By heating the structure, the SMA coating will transform to austenite; therefore, compressive stresses will be induced in the structure, as the SMA coating is trying to change its shape by shrinking.

A key parameter for the coating to obtain a shape memory effect is appropriate aging heat treatment. Ni-Ti coatings of equiatomic composition require high phase transformation temperatures in order to obtain the shape memory effect. On the other hand, the phase transformation temperature of Ni-rich coatings can be tailored depending on the coatings’ microstructure, which depends on the precipitates therein. Such tailoring of the phase transformation temperature of Ni-rich coatings can be achieved by heating during Phase 2 of the aforementioned fabrication process. The exact value of heating temperature depends on the SMA material composition and microstructure. For example, in equiatomic Ni-Ti material, the austenite appears at a phase transformation temperature of about 107 °C. However, for as-received SMAs with Ni-rich composition, in absence of precipitates, the austenitic temperature is 35 °C. Precipitate presence in the material’s microstructure influences the austenitic temperature. For instance, in Ni-rich composition, with incoherent precipitates and austenite matrix of heterogeneous distribution (average particle size of about one micrometer), the austenitic temperature is above 35 °C, while in Ni-rich composition, in which coherent nano-precipitates of homogeneous distribution are formed, the same temperature drops below 35 °C.

Four different aging heat treatments were considered in this study for introducing Ni_4_Ti_3_ precipitates of different sizes in Ni_50.8_Ti_49.2_ (at %) SMA specimens:

1. Heat treatment 1 (HT1), consisting of heating the material at 500 °C for one hour, followed by cooling through water quenching (WQ). During this heat treatment, isolated incoherent Ni_4_Ti_3_ precipitates start to form along the grain boundaries.
2. Heat treatment 2 (HT2), which involves heating the material at 500 °C for 24 hours, followed by cooling through WQ. During this heat treatment, incoherent precipitates start growing in size and spread homogeneously across the matrix.
3. Heat treatment 3 (HT3), consisting of heating the material at 500 °C for one hour followed by WQ, then heating at 300 °C for 24 hours and cooling by water quenching. During this heat treatment, coherent nano-precipitates start forming in the matrix and mix with larger in size, incoherent precipitates.
4. Heat treatment 4 (HT4), which involves heating the material at 300 °C for 100 hours, then cooling by WQ. During this heat treatment, evenly distributed fine coherent nano-precipitates form in the matrix.

Transmission electron microscopy (TEM) imaging was performed using a Libra 200 transmission electron microscope (Carl Zeiss AG, Oberkochen, Germany), operated at 200 kV acceleration voltage, to investigate the formation of precipitates. Specimens for TEM investigations were prepared in thin sections of about 50–70 nm in thickness using a Leica Ultramicrotome (Leica Microsystems, Wetzlar, Germany), then placed on copper grids (400 mesh Cu, Agar).

**Figure 5:**
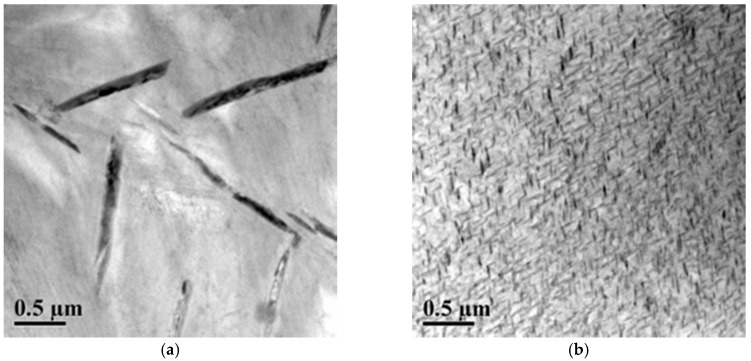
TEM images of Ni-rich Ni-Ti matrix with (**a**) Ni_4_Ti_3_ incoherent precipitates after aging at zero stress at 500 °C for 24 hours and (**b**) Ni_4_Ti_3_ coherent nano-precipitates after aging at zero stress at 300 °C for 100 hours.

Figure 5a presents TEM images of Ni-rich Ni_50.8_Ti (at %) SMA after stress-free aging at 500 °C for 24 hours. It can be observed that Ni_4_Ti_3_ precipitates are incoherent and have rhombohedral atomic structure and lenticular shape [3]. Figure 5b shows TEM images of Ni-rich Ni_50.8_Ti (at %) SMA after stress-free aging at 300 °C for 100 hours. During processing, the Ni-rich Ni-Ti SMA material was subjected to the aforementioned aging heat treatment, in which Ni_4_Ti_3_ coherent nano-precipitates were formed.

## 4. Assessment of Shape Memory Effect of The Ni-Ti Coatings

To determine the transformation heat and the related transformation temperatures of the SMA, Differential Scanning Calorimetry (TA Instruments, New Castle, DE, USA) was used, a method of thermal analysis used to determine phase transformation temperatures of materials, latent heat aroused from transformation, and the specific heat capacity of the material, necessitating tiny quantities of the material [16,19,20]. The principle of operation of DSC method relies on the measurement of the rate that heat energy (heat flow) is provided to a sample to preserve constant heating/cooling rates (power compensated DSC) as a function of temperature and time [1]. 

DSC curves for SMA materials plot the power, in mW, needed to keep constant heating/cooling rates for the SMA sample versus the chamber’s temperature. When the sample is heated from martensitic (twinned) phase, the transformation to austenitic phase starts at the temperature *A_S_*. During reverse transformation, extra heat energy must be provided to the sample to maintain the set heating rate. This energy change provided to the sample as temperature rises is documented as a peak in transformation during heating. A record of an analogous peak is also kept during the cooling route from austenitic to martensitic phase. The transformation temperatures can be measured from the obtained data by drawing tangents at start and finish areas of transformation peaks and the reference of heating/cooling curves. Computation of the specific heat capacity is possible by normalizing the power using the rate of heating and the weight of the sample. The related latent heat for the phase transformation is determined by integration of the specific heat over the transformation temperatures range [1]. Any stored mechanical energy in the sample, such as dislocations introduced by processing, precipitates, and detwinning, can considerably affect the transformation temperatures, thereby making important the starting state of the SMA material. The mechanical energy that is stored in the material can cause shifting or widening of the transformation temperatures, or appearance of an intermediate phase transformation [1].

NiTi SMAs exhibit shape memory effect based on the thermoelastic martensite transformation occurring during cooling and the reverse transformation occurring during heating. Hence, the DSC analysis also allows assessment of the obtained SME. A differential scanning calorimeter from TA Instruments, Q Series, was used to precisely determine the transformation temperatures. The temperature range of the instrument is −180 °C to 600 °C, while inert atmosphere was not used, and cooling was performed using liquid nitrogen. The heating and cooling rates of the tests were fixed to 10 °C/min. The calibration procedure for the temperature scale was performed using the control software according to TA Instruments specifications for the Q Series model and the selected heat flow. The calibration of temperature was based on measuring a temperature standard, indium of high purity, that was heated over its melting point. The recorded melting transition point of the standard was compared to indium’s known melting point, and the difference is considered for the calibration of temperature.

In the tests performed, specimens of 20–40 milligrams Ni-Ti of varying compositions were encapsulated in aluminum pans. The pans’ material was chosen based on the temperature range of the experiment. Aluminum pans are suitable for testing in the range −170 °C to 600 °C. An empty aluminum pan was also inserted in the reference area. Each DSC specimen was tested for three cycles to regulate cycle stability, since the 1st cycle in the tests may present fluctuations due dislocation presence.

**Figure 6:**
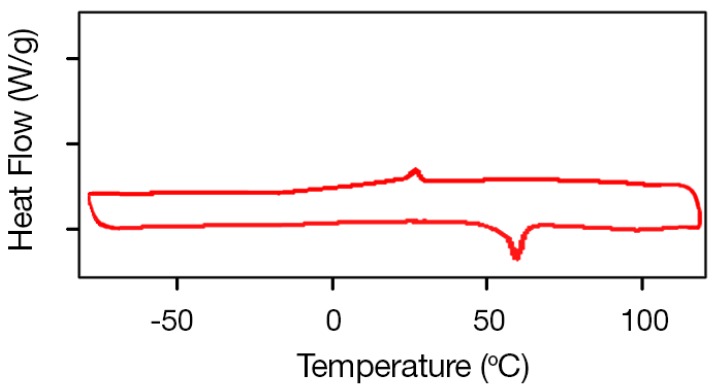
DSC curves of equiatomic (Ni_50_Ti_50_ at. %) SMA.

As mentioned previously, the transformation temperatures of Ni-Ti SMAs strongly depend on alloy composition, in particular at the Ni-rich side. The DSC curve for equiatomic Ni-Ti (Ni_50_Ti_50_ at. %) SMAs, which does not form precipitates when heat treated, is presented in Figure 6 and exhibits peaks revealing transformation temperatures of *M_S_* = 50.8 °C, *M_f_* = 38.3 °C, *A_S_* = 65.4 °C, and *A_f_* = 82.7 °C.

**Figure 7:**
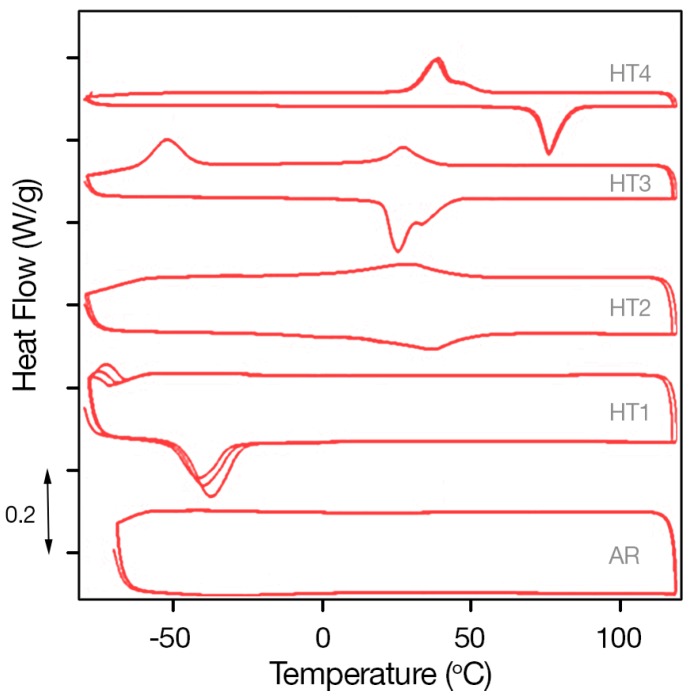
DSC curves of Ni_50.8_Ti_49.2_ (at %) SMA.

The effect of the temperature of aging on the transformation temperatures of the Ni_50.8_Ti_49.2_ (at %) SMAs is shown in the DSC curves of Figure 7, which shows the heating (bottom lines) and cooling (top lines) parts of the analysis of Ni-rich SMAs of Ni_50.8_Ti_49.2_ (at %) SMAs of varying aging heat treatments. The typical DSC curve of an unaged specimen is also included in the figure as a bottom graph, to facilitate comparison with the aged specimens.

As can be observed in Figure 7-AR (bottom plot), no transformation peaks were identified for the unaged/as received (AR) Ni_50.8_Ti_49.2_ SMA. However, observing the DSC plots for heat treatment times/temperatures increasing from HT1 to HT4, an increase in the transformation temperatures is noticed. This is due to the formation of larger precipitates that are depleting nickel from the matrix. The plot associated with heat treatment HT1 (second plot from bottom) shows the DSC response of the Ni_50.8_Ti_49.2_ SMA specimen. The response of this specimen was stabilized after the third temperature cycle. The sample exhibited low transformation peaks relevant with transformation temperatures of *M_S_* = −66.6 °C, *M_f_* = −77.3 °C, *A_S_* = −48.5 °C, *A_f_* = −30.7 °C.

In contrast, sample HT2 exhibits multiple, barely visible transformation peaks at *M_S_* = −49.5 °C and 23.2 °C, *M_f_* = −47.6 °C and 22.8 °C, *A_S_* = 40.8 °C, and *A_f_* = 50.9 °C. Additionally, peak widening can be observed, which is attributed to the formation of incoherent precipitates.

Similarly, multiple wide transformation peaks appear in the plot of sample HT3. The appearance of closely located wide peaks shows that the transformation occurs in two steps rather than in a single step and further supports the reasoning of existence of incoherent precipitates, together with scattered coherent nano-precipitates.

Finally, in the plot for HT4, clear DSC peaks attributed to homogeneous coherent nano-precipitates’ presence can be observed at transformation temperatures of *M_S_* = 47.2 °C, *M_f_* = 29.7 °C, *A_S_* = 68.5 °C, and *A_f_* = 85.0 °C.

Figure 7 shows how aging heat treatment and the subsequent formation of Ni_4_Ti_3_ precipitates in Ni_50.8_Ti_49.2_ SMAs influence the forward and reverse phase transformation temperatures, through the shift of A_f_ and M_f_. It can be observed that the transformation temperatures increase with aging time and temperature. Depending on heat treatment, the transformation temperatures in Ni_50.8_Ti_49.2_ SMAs can pass from negative to positive values, slightly above room temperatures. This is vital information for use of SMA materials in realistic applications such as in SMA coatings on metallic structural substrates proposed in the present study.

**Figure 8:**
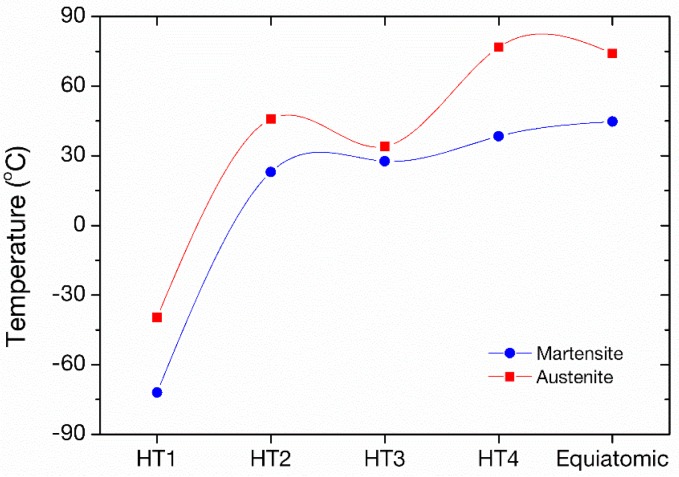
Comparison diagram for the peak transformation temperatures of Ni_50.8_Ti_49.2_ in four different aging heat treatments and of equiatomic Ni-Ti SMA, as per Figure 7.

Figure 8 summarizes the peak temperatures of equiatomic and Ni-rich Ni_50.8_Ti_49.2_ SMA in four different heat treatments, HT1-HT4, for increasing aging temperature and time. The elevated transformation temperatures are observed for the aging heat treatment with the highest temperature/time, which is comparable to the peak temperatures of the equiatomic composition. By examination of the data, the optimum aging heat treatment for Ni_50.8_Ti_49.2_ for using the material in SMA coating form can be clearly identified as HT4.

## 5. Discussion and Conclusions

The present work deals with the development, for the first time, of SMA coatings on structural elements, for modular structures made of aluminum beams for aerospace applications, such as the conventional aircraft wing box and fuselage structures. The SMA coating applications considered here entail long aluminum prisms with their lateral surfaces encased in SMA coating. 

The analysis of the SMA coating–aluminum beam assumes long prisms with variable ratios of SMA thickness to smallest dimension of the beam. According to modelling results, when the SMA coating is initially in the austenitic state and deformed together with the aluminum beam upon uniaxial tensile loading, the required stress levels for sufficient phase transformation to take place are too high for practical applications. The reason being that the transformation strains should be matched by elastic straining of the elastic structure. This loading path is thus not considered of value for the mechanical performance of the elastic structure. The loading path that can induce beneficial compressive stresses upon heating in the aluminum beam requires the SMA coating to be in a detwinned martensitic state when the whole structure, i.e., SMA coating–beam, is under zero load; the SMA-coating should be initially oriented martensite with a deformation that can be recovered upon heating and transformation to austenite.

A proposed fabrication method that will result in initially deformed SMA-coating may be deposition on the compressive side of the aluminum beam when under bending at low temperatures. Load removal will create tensile stresses and detwinning on the SMA-coating, resulting in oriented martensite and macroscopic deformation.

SMA-coated aluminum beam simulations were performed under bending to depict the beneficial effect of the initial compressive stresses induced in the aluminum beam on its surface that is under tension during bending. The outcome of the simulations is that the tensile stresses induced in the presence of the SMA coating are reduced by an amount that is approximately equal to the initial compressive stresses. Thus, the induced compressive stresses contribute to the rigidity and the stiffness of the aluminum beam to endure the applied loads in a safe manner and are further expected to enhance their fatigue properties.

Martensitic transformation determines all critical properties of SMAs, including superelasticity and SME. The final transformation temperature is considered as one of the most significant material properties in Ni-Ti SMAs, since it governs the transition between superelastic and shape memory behavior. The transformation temperature in SMAs can be controlled by choosing proper alloy composition and appropriate aging heat treatment, which modifies the microstructure through introduction of precipitates. Even short-time heat treatments at moderate temperatures can influence the mechanical properties and transformation behavior of SMAs.

Appropriate aging heat treatment was identified as of paramount importance for the coating to exhibit the shape memory effect. Ni-rich Ni_50.8_Ti (at %) SMAs, after stress-free aging, lead to the formation of Ni_4_Ti_3_ precipitates, which directly influences the SMA coating transformation temperature.

For the SMA coating to exhibit shape memory effect, the coating should be subjected to appropriate processing conditions and deposited on a metallic substrate with proper geometry according to the newly suggested two-phase coating deposition process that involves deposition of the coating on the compressive side of a bent aluminum substrate beam. At the final stage, heating the structure will lead the SMA coating to transform to austenite, inducing compressive stresses, as the SMA coating is trying to change its shape by shrinking.

## Figures and Tables

**Table 1:** SMA parameter values.

Parameter	Value	Parameter	Value
*E_A_* [GPa]	33	*H_sat_*	0.025
*E_M_* [GPa]	15	*M^f^* [K]	227
*ν_A_* = *ν_Μ_*	0.33	*M^s^* [K]	242
-	-	*A_s_* [K]	261
-	-	*A_f_* [K]	270
-	-	*C_A_* [MPa K^−1^]	4.5
-	-	*C_M_* [MPa K^−1^]	4.5

Where, *E_A_*, *ν_A_* is the elastic modulus and poisson’s ratio of austenite, respectively; *E*_M_, *ν*_M_ is the elastic modulus and poisson’s ratio of martensite, respectively; *H_sat_* is the maximum attainable transformation strain; *M_f_* is the martensitic finish temperature at zero stress; *M_S_* is the martensitic start temperature at zero stress; *A_S_* is the austenitic start temperature at zero stress; *A_f_* is the austenitic start temperature at zero stress; *C_A_* is the stress influence coefficient of austenite; and *C_M_* is the stress influence coefficient of martensite.

**Table 2:** Elastic properties of aluminum.

Parameter	Value
*E* [GPa]	68.9
*ν*	0.33
